# Effects of increasing soybean meal in late nursery, grower, and finishing pig diets

**DOI:** 10.1093/tas/txag082

**Published:** 2026-06-20

**Authors:** Jamil E G Faccin, Julian Arroyave, Robert D Goodband, Mike D Tokach, Joel M DeRouchey, Jason C Woodworth, Katelyn N Gaffield, Jordan T Gebhardt, Hari B Krishnan

**Affiliations:** Department of Animal Sciences and Industry, College of Agriculture, Kansas State University, Manhattan, KS, 66506-0201, United States; Department of Animal Sciences and Industry, College of Agriculture, Kansas State University, Manhattan, KS, 66506-0201, United States; Department of Animal Sciences and Industry, College of Agriculture, Kansas State University, Manhattan, KS, 66506-0201, United States; Department of Animal Sciences and Industry, College of Agriculture, Kansas State University, Manhattan, KS, 66506-0201, United States; Department of Animal Sciences and Industry, College of Agriculture, Kansas State University, Manhattan, KS, 66506-0201, United States; Department of Animal Sciences and Industry, College of Agriculture, Kansas State University, Manhattan, KS, 66506-0201, United States; Department of Animal Sciences and Industry, College of Agriculture, Kansas State University, Manhattan, KS, 66506-0201, United States; Department of Diagnostic Medicine/Pathobiology, College of Veterinary Medicine, Kansas State University, Manhattan, KS, 66506-0201, United States; US Department of Agriculture-Agricultural Research Service, Plant Genetics Research Unit, Columbia, MO, 65211, United States

**Keywords:** amino acids, crude protein, grow-finishing, nursery, soybean meal, swine

## Abstract

Six experiments were conducted to determine the effects of increasing soybean meal (SBM) in late nursery and finishing pig diets on growth performance, fecal characteristics, and carcass traits. In Exp. 1, 266 pigs (initially 10.1 ± 0.17 kg) were randomly assigned to one of four corn-based diets with SBM levels of 25.0, 28.9, 32.5, or 36.2%. In Exp. 2, 340 pigs (initially 13.5 ± 0.18 kg) were randomly assigned to one of five corn-based diets with SBM levels of 25.0, 28.9, 32.5, 36.2, or 40.0%. In Exp. 1, increasing SBM from 25.0 to 36.2%, decreased (linear, *P* < 0.05) ADG, ADFI, and final BW, with no changes in G:F. In Exp. 2, increasing SBM from 25.0 to 40.0%, decreased ADFI (linear, *P* = 0.017). However, no effect of the SBM level was observed for ADG, final BW, or G:F. In Exp. 3, 615 pigs (initially 43.2 ± 0.68 kg) were randomly assigned to one of five corn-based diets with SBM levels of 19.1, 22.6, 26.3, 29.9, or 33.5% in a 28-d study. At the conclusion of the study, pigs were fed a common diet for 30 d, then re-allotted to one of five corn-based diets with SBM levels of 11.2, 14.2, 17.2, 20.2, or 23.2% (Exp. 4, initially 102.3 ± 1.55 kg). In Exp. 5, 1053 pigs (initially 39.1 ± 0.78 kg) were randomly assigned to one of five corn-based diets containing 20% of corn dry distillers grain with solubles (DDGS) with SBM levels of 18.2, 23.5, 28.9, or 34.3% in a 28-d study. At the conclusion of the study, pigs were fed a common diet for 30 d, then re-allotted (initially 102.2 ± 1.13 kg) to one of five corn-based diets containing 10% DDGS with SBM levels of 9.5, 13.5, 17.5, or 21.5%. For the grower phase, in Exp. 3, increasing SBM increased (linear, *P* = 0.05) ADG and G:F. However, when DDGS were included in the diet (Exp. 5), no differences were observed in growth performance. For late-finishing pigs (Exp. 4 and 6), no differences were observed for any growth performance or carcass criteria when increasing SBM in either corn or corn-DDGS-based diets. In conclusion, increasing SBM decreased growth performance in 10 kg nursery pigs but not in pigs initially 13.5 kg. In the grower phase, increasing SBM improved growth performance in corn-SBM-based diets, but not diets containing DDGS. During the late finishing phase, SBM level did not affect growth performance or carcass characteristics, regardless of diet formulation strategy.

## Teaser

Feeding increasing levels of soybean meal does not affect performance or carcass characteristics of early- and late finishing pigs.

## Introduction

Soybean-derived biodiesel has greater energy potential and lower greenhouse gas emissions than bioethanol and biogas, which has encouraged the United States Renewable Fuel Standard Program to stimulate soybean oil extraction infrastructure in the United States (Esteves et al. 2021; U.S. EPA 2022). Increased soybean oil production will consequently increase the availability of soybean oil co-products, such as soybean meal (**SBM**), in the market. This increased supply may reduce SBM prices, potentially allowing greater utilization by the livestock industry.

Soybean meal is one of the primary plant-based protein sources used in swine diets worldwide and is the most widely used protein source in the United States (Shelton et al. 2001; Sotak-Peper et al. 2017). Its amino acid profile and high digestibility (NRC 2012) allow it to meet nitrogen and amino acid requirements from nursery through finishing phases. In addition to contributing nitrogen compounds, other nutritional properties of SBM may also be relevant when considering increased inclusion rates in swine diets. Recent research indicates that the energy content of SBM is greater than previously estimated (Lopez et al. 2020; Cemin et al. 2020b). Furthermore, SBM contains bioactive compounds such as isoflavones, saponins, peptides, and omega-3 fatty acids, which may confer additional benefits to pigs, particularly under health-challenged conditions (Omoni and Aluko 2005; Smith and Dilger 2018).

Despite its nutritional and functional benefits, the inclusion of SBM in swine diets may be limited by physiological constraints and the presence of antinutritional factors. Nursery pigs, in particular, may have a reduced capacity to digest diets with high crude protein (**CP**) concentrations due to immature proteolytic enzyme systems. This can result in increased amounts of undigested protein reaching the large intestine, where it serves as a substrate for pathogenic bacteria proliferation (Jeaurond et al. 2008). In addition to digestive limitations, antinutritional components in SBM such as trypsin inhibitors may further restrict inclusion rates, especially during early life stages (Miller et al. 2025). While standard thermal processing effectively deactivates these trypsin inhibitors, α-galactosides such as raffinose and stachyose remain highly heat-stable (Karr-Lilienthal et al. 2005). These oligosaccharides restrict SBM utilization in early nursery diets because the immature monogastric gut lacks the endogenous α-1,6-galactosidase enzyme required to digest them, resulting in elevated digesta osmolarity and an accelerated passage rate (Lallès et al. 2007). Ultimately, the accumulation of these undigested carbohydrates in the lower gastrointestinal tract induces local intestinal inflammation and compromises mucosal morphology, which disrupts nutrient absorption, reduces overall energy and protein digestibility, and drives the depressed growth performance in weaned pigs (Li et al. 1990; Smiricky et al. 2002).

Therefore, the objective of the current study was to evaluate the effect of increasing SBM levels in late nursery and finishing pigs on growth performance, fecal dry matter (**DM**), and carcass composition. It was hypothesized that increasing SBM inclusion would maintain growth performance and carcass characteristics, while fecal DM in nursery pigs would worsen as SBM inclusion increased.

## Materials and methods

### General

The Kansas State University Institutional Animal Care and Use Committee approved the protocols used in these experiments (IACUC # 4485.25 and 4375.18). Experiments 1 to 4 were conducted at the Kansas State University Swine Teaching and Research Center in Manhattan, KS. For Exp. 1 and 2, each pen (1.2 × 1.5 m) provided approximately 0.36 m^2^ per pig and was equipped with a 4-hole dry feeder and nipple drinker to provide ad libitum access to feed and water. Facilities had metal tri-bar flooring and were mechanically ventilated with room temperatures set at 32.2°C during the first 10 d and then gradually reduced by 0.28°C daily until the set point reached 25°C. For Exp. 3 and 4, each pen (2.5 × 3 m) provided approximately 0.74 m^2^ per pig and was equipped with a 2-hole dry feeder and a cup drinker to provide ad libitum access to feed and water. Facilities had concrete slat flooring and were mechanically ventilated with room temperatures set at 21°C. Experiments 5 and 6 were conducted at a commercial research-finishing site located in southwest Minnesota. Each pen (3.1 × 5.8 m) provided approximately 0.67 m^2^ per pig and was equipped with a 5-hole dry feeder and a cup drinker to provide ad libitum access to feed and water. Facilities were double-curtain-sided with concrete slat flooring and naturally ventilated.

### Chemical analysis

Representative samples of SBM were analyzed at the University of Missouri Agricultural Experiment Station Chemical Laboratory for CP (Table 1; Method 990.03; AOAC International 2006), ash, DM (Method 935.29; AOAC International, 2006), ether extract (ANKOM Technology 2004), and NDF (ANKOM Technology 2005). Samples were also submitted to the USDA, ARS, Plant Genetic Resources Unit, University of MO for trypsin inhibitor units (Kim and Krishnan 2023).

**Table 1:** Chemical analysis of soybean meal, as-fed basis^a^.

Item, %	Experiment		
	1 to 4	5	6
**Dry matter**	87.71	87.82	88.07
**Crude protein**	46.22	47.55	46.99
**Ash**	6.26	6.12	6.24
**Ether extract**	2.12	2.11	2.26
**Neutral detergent fiber**	8.39	6.32	7.25
**Trypsin inhibitor units/mg**	5.04	4.03	3.06

a A representative sample of each soybean meal source was obtained, homogenized, and submitted to the University of Missouri Agricultural Experiment Station Chemical Laboratory, Columbia MO, for proximate analysis and to USDA, ARS, Plant Genetic Resources Unit (University of Missouri) for trypsin inhibitor analysis.

### Animals and diets

*Late nursery phase*: In Exp. 1, a total of 266 pigs (241 × 600, DNA; initially 10.1 ± 0.17 kg and 40 d of age) were used in a 21-d trial with 14 replicate pens per treatment and 4 to 5 pigs per pen. Pens of pigs were randomly assigned to one of four dietary treatments containing increased levels of SBM (25.0, 28.9, 32.5, or 36.2%; Table 2). In Exp. 2, a total of 340 pigs (initially 13.5 ± 0.18 kg and 45 d of age) were used in a 21-d trial with 14 replicate pens per treatment and 4 to 5 pigs per pen. Pens of pigs were randomly assigned to one of five dietary treatments containing increased levels of SBM (25.0, 28.9, 32.5, 36.2, or 40.0%; Table 2). At weaning, pigs were assigned to pens balanced by age, body weight (*BW*), gender, and sow parity. On d 21 (Exp. 1) and d 26 (Exp. 2) post-placement, pens were randomly assigned to dietary treatments using a randomized complete block design, with initial BW serving as the blocking factor.

**Table 2:** Diet composition, Exp. 1 and 2 (as-fed basis).

	Soybean meal, %				
Item	25.0	28.9	32.5	36.2	40.0^a^
**Ingredient, %**					
** Corn**	69.50	66.05	62.73	59.36	55.88
** Soybean meal (46.2% CP)**	25.02	28.86	32.53	36.20	40.04
** Soybean oil**	1.00	1.00	1.00	1.00	1.00
** Calcium carbonate**	0.73	0.75	0.75	0.78	0.78
** Monocalcium P (21% P)**	1.10	1.05	1.00	0.95	0.90
** Sodium chloride**	0.60	0.60	0.60	0.60	0.60
** L-Lys-HCl**	0.65	0.53	0.42	0.30	0.18
** DL-Met**	0.28	0.24	0.21	0.18	0.14
** L-Trp**	0.09	0.07	0.05	0.03	–
** L-Thr**	0.30	0.25	0.20	0.15	0.10
** L-Val**	0.25	0.19	0.13	0.06	–
** L-Ile**	0.09	0.03	–	–	–
** Vitamin premix with phytase^b^**	0.25	0.25	0.25	0.25	0.25
** Trace mineral premix**	0.15	0.15	0.15	0.15	0.15
**Calculated analysis**					
**Standardized ileal digestible (SID) AA, %**					
** Lys**	1.30	1.30	1.30	1.30	1.30
** Ile:Lys**	56	56	59	64	69
** Leu:Lys**	106	113	120	127	134
** Met:Lys**	40	39	38	37	35
** Met and Cys:Lys**	60	60	60	60	60
** Thr:Lys**	65	65	65	65	65
** Trp:Lys**	20.6	20.6	20.6	20.6	20.6
** Val:Lys**	73	73	73	73	73
** His:Lys**	33	36	38	41	44
**SID Lys:Net Energy, g/Mcal**	4.88	4.89	4.89	4.90	4.90
**Net Energy, kcal/kg^c^**	2,661	2,659	2,657	2,654	2,652
**SID Lys:CP, %**	6.84	6.43	6.04	5.73	5.42
**Ca, %**	0.65	0.66	0.66	0.67	0.68
**STTD P, %**	0.47	0.47	0.47	0.47	0.47
**CP, %**	19.0	20.2	21.5	22.7	24.0
**NDF, %**	8.4	8.4	8.4	8.4	8.4

a Treatment with 40.0% SBM was only used in Exp. 2.

b Ronozyme HiPhos GT 2700 (DSM Nutritional Products, Parsippany, NJ) at 560 FTU/kg with an expected STTD P release of 0.12%.

c Soybean meal was assumed to have 100% NE as corn.

CP, crude protein.

STTD, standardized total tract digestible.

*Grower and late finishing phase*: In Exp. 3, a total of 615 pigs (43.2 ± 0.69 kg) were used in a 28-d trial with 14 replicate pens per treatment and 8 to 10 pigs per pen. Pens were randomly assigned to one of five dietary treatments containing increasing levels of SBM (19.1, 22.6, 26.3, 29.9, or 33.5%; Table 3) in a corn-based diet. At the conclusion of the study, all pigs were fed a common diet for 30 days (flushing period). In Exp. 4, the same 615 pigs (102.3 ± 1.55 kg) were used in a 30-d experiment with 14 replicate pens per treatment and 8 to 10 pigs per pen. Pens were randomly assigned to one of five dietary treatments containing increased levels of SBM (11.2, 14.2, 17.2, 20.2, or 23.2%; Table 4) in a corn-based diet.

**Table 3:** Diet composition, Exp. 3 (as-fed basis).

	Soybean meal, %^a^	
Item	19.1	33.5
**Ingredient, %**		
** Corn**	77.44	64.25
** Soybean meal (46.2% CP)**	19.06	33.45
** Calcium carbonate**	0.90	0.90
** Monocalcium P (21% P)**	0.80	0.60
** Salt**	0.50	0.50
** L-Lys-HCl**	0.45	–
** DL-Met**	0.14	–
** L-Trp**	0.06	–
** L-Thr**	0.19	–
** L-Val**	0.11	–
** L-Ile**	0.06	–
** Vitamin premix with phytase^b^**	0.15	0.15
** Trace mineral premix**	0.15	0.15
**Calculated analysis**		
**Standardized ileal digestible (SID) AA, %**		
** Lys**	1.00	1.00
** Ile:Lys**	60	78
** Leu:Lys**	125	160
** Met:Lys**	37	29
** Met and Cys:Lys**	60	59
** Trp:Lys**	21.0	23.2
** Thr:Lys**	65	67
** Val:Lys**	72	85
**SID Lys:Net Energy, g/Mcal**	3.82	3.83
**Net Energy, kcal/kg^c^**	2,615	2,610
**SID Lys:CP, %**	6.13	4.69
**Ca, %**	0.62	0.63
**STTD P, %**	0.39	0.39
**Chemical analysis, %**		
** CP, %**	16.3	21.3
** NDF, %**	8.6	8.6

a Diets with 19.1 and 33.5% SBM were blended to provide the 3 intermediate treatment levels.

b Ronozyme HiPhos GT 2700 (DSM Nutritional Products, Parsippany, NJ) at 560 FTU/kg with an expected STTD P release of 0.12%.

c Soybean meal was assumed to have 100% NE as corn.

CP, crude protein.

STTD, standardized total tract digestible.

**Table 4:** Diet composition, Exp. 4 (as-fed basis).

	Soybean meal, %^a^	
Item	11.2	23.2
**Ingredient, %**		
** Corn**	86.23	75.14
** Soybean meal (46.2% CP)**	11.20	23.19
** Calcium carbonate**	0.75	0.75
** Monocalcium P (21% P)**	0.30	0.12
** Sodium chloride**	0.50	0.50
** L-Lys-HCl**	0.38	–
** DL-Met**	0.06	–
** L-Trp**	0.05	–
** L-Thr**	0.14	–
** L-Val**	0.06	–
** L-Ile**	0.05	–
** Vitamin premix with phytase^b^**	0.15	0.15
** Trace mineral premix**	0.15	0.15
**Calculated analysis**		
**Standardized ileal digestible (SID) AA, %**		
** Lys**	0.75	0.75
** Ile:Lys**	61	82
** Leu:Lys**	144	182
** Met:Lys**	34	33
** Met and Cys:Lys**	60	67
** Trp:Lys**	21.0	23.4
** Thr:Lys**	67	70
** Val:Lys**	72	91
**SID Lys:Net Energy, g/Mcal**	2.85	2.85
**Net Energy, kcal/kg^c^**	2,630	2,628
**SID Lys:CP, %**	5.73	4.34
**Ca, %**	0.45	0.46
**STTD P, %**	0.28	0.28
**Chemical analysis, %**		
**CP, %**	13.1	17.3
**NDF, %**	8.6	8.6

a Diets with 11.2 and 23.2% SBM were blended to match the 3 intermediate treatment levels.

b Ronozyme HiPhos GT 2700 (DSM Nutritional Products, Parsippany, NJ) at 560 FTU/kg with an expected STTD P release of 0.12%.

c Soybean meal was assumed to have 100% NE as corn.

CP, crude protein.

STTD, standardized total tract digestible.

In Exp. 5, a total of 1,053 pigs (initially 39.1 ± 0.78 kg) were used in a 28-d trial with 10 replicate pens per treatment and 27 pigs per pen. Pens of pigs were assigned to one of four dietary treatments containing increasing levels of SBM (18.2, 23.5, 28.9, or 34.3%; Table 5) in a corn-based diet containing 20% of corn dried distillers grain with solubles (*DDGS*). At the conclusion of the study, all pigs were fed a common diet for 30 d (flushing period). In Exp. 6, the same pens of pigs (102.2 ± 1.13 kg) were used in a 33-d trial with 10 replicate pens per treatment and 27 pigs per pen. Pens were randomly assigned to one of four dietary treatments containing increasing levels of SBM (9.5, 13.5, 17.5, or 21.5%; Table 6) in a corn-based diet containing 10% corn DDGS.

**Table 5:** Diet composition, Exp. 5 (as-fed basis).

	Soybean meal, %			
Item	18.2	23.5	28.9	34.3
**Ingredient, %**				
** Corn**	58.50	53.51	48.54	43.55
** Soybean meal (47.6% CP)**	18.16	23.53	28.88	34.25
** DDGS**	20.00	20.00	20.00	20.00
** Calcium carbonate**	1.20	1.20	1.20	1.20
** Monocalcium P (21% P)**	0.55	0.47	0.40	0.33
** Sodium chloride**	0.50	0.50	0.50	0.50
** L-Lys-HCl**	0.50	0.33	0.17	–
** DL-Met**	0.10	0.06	0.03	–
** L-Trp**	0.05	0.03	0.02	–
** Thr^a^**	0.19	0.12	0.06	–
** L-Val**	0.09	0.06	0.03	–
** Tribasic copper chloride**	0.03	0.03	0.03	0.03
** Phytase^b^**	0.05	0.05	0.05	0.05
** Vitamin-trace mineral premix**	0.10	0.10	0.10	0.10
**Calculated analysis**				
**Standardized ileal digestible (SID) AA, %**				
** Lys**	1.10	1.10	1.10	1.10
** Ile: Lys**	58	66	74	82
** Leu: Lys**	145	157	169	180
** Met: Lys**	35	34	34	33
** Met and Cys: Lys**	60	62	63	65
** Trp: Lys**	19.8	21.0	22.2	23.4
** Thr: Lys**	65	67	69	71
** Val: Lys**	75	80	86	92
**SID Lys: Net Energy, g/Mcal**	4.38	4.38	4.38	4.38
**Net Energy, kcal/kg^c^**	2,513	2,513	2,511	2,511
**SID Lys: CP, %**	5.53	5.05	4.64	4.30
**Ca, %**	0.61	0.61	0.62	0.62
**STTD P, %**	0.45	0.45	0.45	0.45
**Chemical analysis, %**				
** CP, %**	19.9	21.8	23.7	25.6
** NDF, %**	12.2	12.2	12.2	12.2

a ThrPro (CJ America-Bio, FortDodge, IA).

b Optiphos Plus 2500 G (Huvepharma Inc. Peachtree City, GA) at 700 FTU/kg with an expected STTD P release of 0.13%.

c Soybean meal was assumed to have 100% NE as corn.

CP, crude protein.

STTD, standardized total tract digestible.

**Table 6:** Diet composition, Exp. 6 (as-fed basis).

	Soybean meal, %			
Item	9.5	13.5	17.5	21.6
**Ingredient, %**				
** Corn**	77.99	74.20	70.45	66.66
** Soybean meal (47.0% CP)**	9.49	13.52	17.53	21.57
** DDGS**	10.00	10.00	10.00	10.00
** Calcium carbonate**	0.95	0.95	0.95	0.95
** Monocalcium P (21% P)**	0.30	0.26	0.22	0.18
** Sodium chloride**	0.50	0.50	0.50	0.50
** L-Lys-HCl**	0.38	0.25	0.13	–
** DL-Met**	0.05	0.03	0.02	–
** L-Trp**	0.04	0.03	0.01	–
** Thr^a^**	0.14	0.09	0.05	–
** L-Val**	0.03	0.02	0.01	–
** Tribasic copper chloride**	0.03	0.03	0.03	0.03
** Phytase^b^**	0.02	0.02	0.02	0.02
** Vitamin-trace mineral premix**	0.10	0.10	0.10	0.10
**Calculated analysis**				
**Standardized ileal digestible (SID) AA, %**				
** Lys**	0.75	0.75	0.75	0.75
** Ile:Lys**	58	67	76	85
** Leu:Lys**	162	175	188	201
** Met:Lys**	36	36	36	37
** Met and Cys:Lys**	65	67	70	72
** Trp:Lys**	19.9	21.1	22.4	23.6
** Thr:Lys**	67	69	72	75
** Val:Lys**	75	82	90	97
**SID Lys:Net Energy, g/Mcal**	2.91	2.91	2.91	2.91
**Net Energy, kcal/kg^c^**	2,575	2,575	2,575	2,573
**SID Lys:CP, %**	5.24	4.78	4.36	4.03
**Ca, %**	0.45	0.45	0.46	0.46
**STTD P, %**	0.32	0.33	0.33	0.34
**Chemical analysis, %**	5.24	4.78	4.36	4.03
** CP, %**	14.3	15.7	17.2	18.6
** NDF, %**	10.6	10.6	10.6	10.5

a ThrPro (CJ America-Bio, FortDodge, IA).

b Optiphos Plus 2500 G (Huvepharma Inc. Peachtree City, GA) at 700 FTU/kg with an expected STTD P release of 0.13%.

c Soybean meal was assumed to have 100% NE as corn.

CP, crude protein.

STTD, standardized total tract digestible.

In all experiments, dietary treatments were formulated by replacing feed-grade amino acids (*AA*) with increasing dietary SBM. Within each experiment, diets were formulated to maintain the same minimum standardized ileal digestible lysine *(SID Lys*), amino acid ratios relative to Lys, and similar energy content; however, CP increased with higher SBM inclusion, resulting in a reduction of SID Lys relative to CP as SBM levels increased. The initial low SBM diets were formulated to contain a minimum ratio of SID AA relative to Lys that was above NRC (2012) requirement estimates so that the increasing AA: Lys ratios would not be a factor in the response criteria. Experimental diets were formulated to the fifth or sixth limiting AA with the addition of feed-grade amino acids. The degree of substitution with feed-grade AA was based on practical applications that might be used in diet formulation based on commercially available AAs. Diets used in Exp. 1 and 2 were manufactured at Hubbard Feeds in Beloit, KS. For Exp 3, 4, 5, and 6, diets with the lowest and highest amount of SBM were manufactured and intermediate SBM levels were achieved by blending these diets using a robotic feeding system (FeedPro; Feedlogic Corp., Wilmar, MN). The same feeding system was used to record feed deliveries for individual pens. All diets used in these studies were fed in meal form.

### Experimental procedures

For the late nursery experiments, pen weights and feed disappearance were measured on d 0, 7, 14, and 21 of the experiment to determine average daily gain (*ADG*), average daily feed intake (*ADFI*), and feed efficiency (*G:F*). Fecal samples, free catch, were collected on d 7 and 21 from the same three medium weight pigs in each pen. After collection, fecal samples were dried at 55°C in a forced air oven (Model SMO28-2, Shel Lab, Cornelius, OR) for 48 h and the ratio of dried to wet fecal weight determined percentage fecal DM.

In the grower and late finishing experiments, pigs were weighed at allotment, at the midpoint of the experiment (d 14), and at the conclusion of each trial (approximately 28 d after the trial started). In Exp. 6, the three heaviest pigs in each pen were selected and marketed 21-d before the conclusion of the study but not included in the final pen carcass data. In Exp. 4 and 6, on the last day of the trial, final pen weights were obtained, and all pigs were tattooed with a pen identification number and transported to a USDA-inspected packing plant (JBS, Worthington, MN) for carcass data collection. The pigs used for carcass data collection were slaughtered on the same day. Carcass measurements included hot carcass weight (*HCW*), loin depth, backfat depth, and percentage lean. Percentage lean was calculated from a proprietary plant equation. Carcass yield was calculated by dividing the pen average HCW by the pen average final live weight obtained at the farm.

### Statistical analysis

Data were analyzed using the GLIMMIX procedure of SAS version 9.4 (SAS Institute, Inc., Cary, NC) in a randomized complete block design for Exp. 1 and 2, and as a completely randomized design for Exp. 3, 4, 5, and 6 with pen serving as the experimental unit in all experiments. The initial BW was tested as a covariate and was used when it reduced the Bayesian Information Criteria by at least 2 units. The statistical model considered the fixed effect of dietary treatment and, for Exp 1 and 2, the random effect of the block. Contrasts were used to test for the linear and quadratic response of different SBM levels. In Exp. 1 and 2, fecal DM was analyzed as repeated measures, and pen was included in the model as a random effect to account for subsampling attributed to the multiple observations for each pen. Level of SBM, day, and the associated interactions were considered fixed effects within the statistical model. In Exp. 4 and 6, HCW served as a covariate for backfat, loin depth, and lean percentage. Results from the experiment were considered significant at *p* < 0.05 and marginally significant between *p* > 0.05 and *p* ≤ 0.10.

## Results

### Late nursery phase (Exp. 1 and 2)

In Exp. 1, for the overall 21-d period, increasing SBM from 25.0 to 36.2% decreased ADG (linear, *p* = 0.012), ADFI (linear, *p* < 0.001), and d 21 BW (linear, *p* = 0.021; Table 7). No differences were observed for G:F. In Exp. 2, increasing SBM from 25.0 to 40.0% decreased (linear, *p* = 0.017) ADFI; however, no differences were observed for ADG, final BW, or G:F.

**Table 7:** Effects of increasing soybean meal on growth performance and fecal dry matter (DM) of 10 or 13 kg nursery pigs.^a^

	Soybean meal, %						*P* =	
Item	25.0	28.9	32.5	36.2	40.0^b^	SEM	Linear	Quadratic
**Exp. 1**								
** BW, kg**								
** d 0**	10.1	10.1	10.1	10.1	–	0.17	0.956	0.990
** d 21**	22.0	22.9	21.1	21.2	–	0.38	0.021	0.189
** 0 to 21 d**								
** ADG, g**	567	594	522	531	–	15.8	0.012	0.569
** ADFI, g**	857	857	789	780	–	15.9	< 0.001	0.742
** G:F, g/kg**	661	693	661	680	–	11.2	0.975	0.729
** Fecal DM^c^**								
** d 7**	20.6	20.1	19.6	17.5	–	0.40	0.006	0.317
** d 21**	21.3	21.8	20.8	21.7	–	0.40	0.962	0.812
**Exp. 2**								
** BW, kg**								
** d 0**	13.5	13.6	13.5	13.6	13.5	0.18	0.572	0.538
** d 21**	28.1	28.3	28.3	27.9	27.8	0.40	0.441	0.487
** 0 to 21 d**								
** ADG, g**	694	699	708	680	680	13.6	0.295	0.478
** ADFI, g**	1,057	1,039	1,048	1,016	1,007	15.9	0.017	0.800
** G:F, g/kg**	657	672	675	670	676	9.0	0.198	0.379
** Fecal DM^d^**								
** d 7**	20.8	19.6	18.7	17.0	16.6	0.43	< 0.001	0.832
** d 21**	22.0	21.3	22.5	19.3	19.9	0.43	0.046	0.644

a A total of 266 and 340 pigs for experiments 1 and 2, respectively, were used in a 21-d trial. Each pen had 4 to 5 pigs and each treatment had 14 replicate pens. Feces from three piglets from each pen were taken in plastic bags on d 7 and 21, individually weighed, and dried to measure fecal dry matter.

b Treatment with 40.0% soybean meal was only used in Exp. 2.

c Soybean meal × day, linear *p* = 0.030, quadratic *p* = 0.344; Day, *p* < 0.001.

d Soybean meal × day, linear *p* = 0.180, quadratic *p* = 0.377; Day, *p* < 0.001.

For fecal DM, in Exp 1, there was a SBM level × day interaction (linear, *p* = 0.030) where on d 7 increasing SBM decreased (linear, *p* = 0.006) fecal DM but no effect of SBM level was observed on d 21. In Exp 2, no SBM level × day interaction was observed. Increasing SBM decreased fecal DM on d 7 (linear, *p* < 0.001) and d 21 (linear, *p* = 0.046). In both experiments, the main effect of day was observed (*p* < 0.001), with fecal DM being greater on day 21 than on day 7.

### Grower and late finishing phases (Exp 3, 4, 5, and 6)

In Exp. 3, increasing SBM increased (linear, *p* = 0.05) ADG and G:F (Table 8). No differences were observed for final weight or ADFI. However, in diets containing 20% DDGS (Exp. 5; Table 9), regardless of the level of SBM provided, no differences were observed for any growth performance criteria.

**Table 8:** Effects of increasing soybean meal on growth performance of grower pigs (Exp. 3).^a^

	Soybean meal, %						*P* =	
Item	19.1	23.7	26.3	29.9	33.5	SEM	Linear	Quadratic
**BW, kg**								
** d 0**	43.2	43.2	43.2	43.1	43.2	0.68	0.956	0.996
** d 28**	71.2	72.0	71.5	71.8	72.1	0.55	0.415	0.954
**Day 0 to 28**								
** ADG, g**	998	1,030	1,012	1,025	1,034	19.1	0.038	0.747
** ADFI, g**	2,164	2,182	2,136	2,127	2,159	31.3	0.471	0.456
** G:F, g/kg**	461	472	473	482	479	4.2	< 0.001	0.089

a A total of 615 pigs were used in a 28-d trial with 8 to 10 pigs per pen and 14 replicate pens per treatment.

**Table 9:** Effects of increasing soybean meal in diets containing 20% dried distillers grains with solubles on growth performance of grower pigs (Exp. 5).^a^

	Soybean meal, %					*P* =	
Item	18.2	23.5	28.9	34.3	SEM	Linear	Quadratic
**BW, kg**							
** d 0**	39.1	39.1	39.1	39.1	0.78	0.993	0.988
** d 28**	67.0	66.7	67.0	66.5	1.02	0.776	0.886
**Day 0 to 28**							
** ADG, g**	989	984	993	966	12.7	0.321	0.394
** ADFI, g**	2,182	2,159	2,186	2,155	32.7	0.704	0.877
** G:F, g/kg**	453	456	454	448	5.9	0.613	0.431

a A total of 1053 pigs were used in a 28-d trial with 27 pigs per pen and 9 or 10 replicate pens per treatment.

For late-finishing pigs (Exp. 4 and 6), pigs fed SBM levels ranging from 9.5% to 23.2% had similar final weight, ADG, ADFI, and G:F in either corn-based (Table 10) or corn-10% DDGS-based diets (Table 11). Carcass characteristics (HCW, yield, backfat, percentage lean, and loin depth) were not affected by the levels of SBM fed.

**Table 10:** **Effects of increasing soybean meal on growth performance of late finishing pigs (Exp. 4)**.**^a^**

	Soybean meal, %						*P* =	
Item	11.2	14.2	17.2	20.2	23.2	SEM	Linear	Quadratic
**BW, kg**								
** d 0**	102.3	102.3	102.2	102.4	102.3	1.55	0.964	0.981
** d 30**	130.3	131.0	131.0	131.5	130.9	2.15	0.352	0.284
**Day 0 to 30**								
** ADG, g**	930	957	957	966	948	7.2	0.411	0.179
** ADFI, g**	2,771	2,817	2,808	2,776	2,771	35.8	0.758	0.406
** G:F, g/kg^b^**	336	340	341	348	342	15.6	0.115	0.336
**Carcass characteristics**								
** HCW, kg^b^**	97.1	97.4	97.6	97.6	97.0	1.09	0.978	0.384
** Yield, %^c^**	74.4	74.3	74.4	74.2	74.2	0.43	0.311	0.984
** Backfat, mm^c^**	14.7	14.5	14.5	13.9	14.5	0.43	0.132	0.170
** Lean, %^c^**	56.2	55.9	56.0	56.3	55.9	0.21	0.727	0.990
** Loin depth, mm^c^**	67.4	67.6	67.1	67.8	67.6	1.44	0.707	0.809

a A total of 615 pigs were used in a 30-d trial with 8 to 10 pigs per pen and 14 replicate pens per treatment.

b Initial BW was used as a covariate due to lower Bayesian information criterion.

c Carcass characteristics include HCW as a covariate.

**Table 11:** Effects of increasing soybean meal in diets with 10% dried distillers grains with solubles on growth performance and carcass characteristics of late finishing pigs (Exp. 6).^a^

	Soybean meal, %^b^					*P* =	
Item	9.5	13.5	17.5	21.6	SEM	Linear	Quadratic
**BW, kg**							
** d 0**	102.2	102.1	102.2	102.2	1.13	0.989	0.966
** d 33**	126.7	125.7	126.6	126.7	1.36	0.964	0.875
**Day 0 to 33**							
** ADG, g**	798	785	812	798	15.0	0.600	0.421
** ADFI, g**	2,599	2,622	2,608	2,599	25.4	0.720	0.816
** G:F, g/kg^b^**	307	299	311	307	5.1	0.417	0.384
**Carcass characteristics**							
** HCW, kg^b^**	92.6	93.0	92.9	92.6	0.53	0.806	0.642
** Yield, %^c^**	73.0	72.1	72.6	72.6	0.41	0.699	0.271
** Backfat, mm^c^**	15.1	15.0	15.0	14.7	0.41	0.536	0.807
** Lean, %^c^**	57.1	57.2	57.1	57.4	0.24	0.449	0.706
** Loin depth, mm^c^**	62.5	62.8	62.2	63.0	0.61	0.695	0.690

a A total of 1080 pigs were used in a 33-d trial with 27 pigs per pen and 10 replicate pens per treatment.

b Initial BW was used as a covariate due to lower Bayesian information criterion.

c Carcass characteristics include HCW as a covariate.

## Discussion

A possible decrease in SBM cost resulting from increased market availability underscores the need to evaluate optimal SBM inclusion levels in swine diets. Six experiments were conducted to evaluate the effects of increasing SBM inclusion under different diet formulation strategies on growth performance, fecal characteristics, and carcass composition. Based on the negative growth performance responses observed in Exp. 1 when SBM inclusion increased from 25.0% to 36.2%, Exp. 2 was designed to validate these findings and to evaluate an additional, higher SBM inclusion level (40.0%). Experiments 3 and 4 were conducted to evaluate the effects of increasing SBM inclusion in corn-based diets during the grower and late finishing phases, respectively. Finally, because the inclusion of corn DDGS is a common industry practice, Exp. 5 and 6 were designed to evaluate the effects of increasing SBM inclusion in grower and finishing diets containing DDGS.

In Exp. 1, high levels of SBM decreased ADG, despite other studies not finding a reduction in ADG with similar SBM levels (Cemin et al. 2020a). Diets containing high levels of SBM and low levels of feed-grade AA, tend to have high CP levels, which could adversely affect performance (Heo et al. 2013). Undigested protein could serve as a substrate for pathogenic bacteria proliferation in the large intestine, leading to lower fecal DM, and a higher energy cost to metabolize and excrete the extra nitrogen (Halas et al. 2007; Van Milgen and Dourmad, 2015). These factors may explain the decreased growth performance (Exp. 1) as well as differences in fecal DM (Exp. 1 and 2) observed as SBM level increased. Antinutritional factors found in SBM, like trypsin inhibitor, have also been demonstrated to reduce AA bioavailability, negatively affecting pig performance (Goebel and Stein, 2010). Recently, Nisley et al. (2024) and Miller et al. (2025) reported a linear reduction in growth performance as trypsin inhibitor units (*TIU*) increased in nursery diets. The SBM used in the present experiments contained 5.04 TIU/mg, suggesting that increasing SBM inclusion resulted in greater dietary TIU concentrations, which may have contributed to the reduced growth performance, particularly in young pigs (less mature gastrointestinal tract).

The differing ADG responses to increasing SBM levels observed between Exp. 1 and 2 may be partially explained by differences in initial BW and age of the pigs. After weaning, pigs are exposed to new feed ingredients, requiring adaptation of the gastrointestinal tract to efficiently digest complex protein sources such as SBM (Batson et al. 2021). Consequently, nursery diets typically contain low SBM levels during early postweaning phases to mitigate hypersensitivity reactions (Li et al. 1990). The results of the present study suggest that pigs in Exp. 2, which were approximately 3.5 kg heavier and 5 d older at the start of the trial, were better able to tolerate higher SBM inclusion levels than pigs in Exp. 1, resulting in no observed ADG response in Exp. 2 compared with the negative response observed in Exp. 1.

When pigs were fed increasing levels of SBM during the grower phase (Exp. 3 and 5), starting at approximately 40 kg BW, growth performance differed between experiments. In Exp. 3, ADG and G:F increased linearly with increasing SBM, although arguably the largest change occurred as SBM increased from 19.1 to 23.7% of the diet with little change thereafter. This may possibly reflect a response to increasing CP or SID Lys:CP ratio whereas increasing CP would supply greater dietary nitrogen availability supporting nonessential amino acid synthesis (Mansilla et al. 2017).

In Exp 5, the inclusion of 20% DDGS resulted in a dietary CP level of 19.9% for the lowest SBM inclusion, compared to only 16.3% CP in Exp. 3. This may partially explain the lack of performance differences when increasing SBM in the 20%-DDGS diet if 16.3% CP is marginal for 40 kg pigs. Phillips et al. (2026) increased SBM inclusion in corn-based diets for finishing pigs (27.6 to 106.4 kg BW) and reported no overall differences in growth performance.

Pigs in Exp. 3 did not experience any disease challenges (0% mortality) as all animals originated from a single sow farm and were housed within a controlled university research environment. This context suggests that the observed improvements in performance were unlikely to be driven by the nutraceutical effects of SBM, which contrasts with studies by Rochell et al. (2015) and Boyd et al. (2010), where the benefits of increased SBM inclusion were observed primarily in disease-challenged pigs.

In late-finishing pigs (>100 kg), Soto et al. (2019) and Holen et al. (2023) have observed that a minimum of 12% of dietary CP is necessary to maximize growth performance. In the current study, even the diets with the lowest inclusion of SBM provided in Exp 4 and 6 had dietary CP levels above 13%. This suggests that the adequate CP levels may explain the similar performance across SBM levels in both experiments.

Although SBM effects on growth performance are well documented, only a few studies have investigated the effects of different levels of SBM on carcass traits. When replacing SBM with feed-grade AA, Kerr et al. (2003) observed limited effects of SBM on carcass traits. Soto et al. (2019) only observed improvements related to carcass performance, such as HCW and carcass ADG. However, consistent with the current study, no significant effects were observed on carcass yield, backfat thickness, loin depth, or lean percentage. Boyd et al. (2010) observed benefits in carcass performance when feeding over 30% SBM to disease-challenged late-finishing pigs. Nevertheless, SBM levels did not affect carcass yield in their study, which is similar to the findings of the present study.

In conclusion, in 10 kg pigs, increasing SBM inclusion reduced growth performance, whereas there were no effects when pigs were fed the same diets and started on test at 13.5 kg. Collectively, fecal DM worsened as SBM levels increased, especially 7-d post-weaning. In the grower phase, increasing SBM increased ADG and G:F only in corn-based diets but not diets containing DDGS. During the late finishing phase, SBM inclusion did not affect growth performance or carcass characteristics, regardless of diet formulation strategy. In growing and finishing pigs, at least 33.4 and 23.2% soybean meal, respectively, can be used without compromising growth performance or carcass characteristics.

## List of abbreviations

AA: amino acids
ADG: average daily gain
ADFI: average daily feed intake
BW: body weight
DDGS: dried distillers grains with solubles
DM: dry matter
G: F: gain-to-feed ratio
HCW: hot carcass weight
SBM: soybean meal
SID: standardized ileal digestible
TIU: trypsin inhibitor units
